# Theoretical study on photocatalytic performance of ZnO/C_2_N heterostructure towards high efficiency water splitting

**DOI:** 10.3389/fchem.2022.1048437

**Published:** 2022-10-19

**Authors:** Meiping Liu, Yong Tang, Haizi Yao, Liuyang Bai, Jun Song, Benyuan Ma

**Affiliations:** ^1^ Henan Key Laboratory of Smart Lighting, Huanghuai University, Zhumadian, Henan, China; ^2^ School of Materials Science and Engineering, Xiangtan University, Xiangtan, Hunan, China; ^3^ School of Energy Engineering, Huanghuai University, Zhumadian, Henan, China

**Keywords:** ZnO/C_2_N heterostructure, first-principles method, type-II band alignment, water splitting, carrier mobility, strain

## Abstract

The construction of van der Waals heterostructures offers effective boosting of the photocatalytic performance of two-dimensional materials. In this study, which uses the first-principles method, the electronic and absorptive properties of an emerging ZnO/C_2_N heterostructure are systematically explored to determine the structure’s photocatalytic potential. The results demonstrate that ZnO and C_2_N form a type-II band alignment heterostructure with a reduced band gap, and hence superior absorption in the visible region. Furthermore, the band edge positions of a ZnO/C_2_N heterostructure meet the requirements for spontaneous water splitting. The ZnO/C_2_N heterostructure is known to possess considerably improved carrier mobility, which is advantageous in the separation and migration of carriers. The Gibbs free energy calculation confirms the high catalytic activity of the ZnO/C_2_N heterostructure for water-splitting reactions. All the aforementioned properties, including band gap, band edge positions, and optical absorption, can be directly tuned using biaxial lateral strain. A suitable band gap, decent band edge positions, high catalytic activity, and superior carrier mobility thus identify a ZnO/C_2_N heterostructure as a prominent potential photocatalyst for water splitting.

## Introduction

The splitting of water into hydrogen (H_2_) and oxygen (O_2_) under the action of a photocatalyst has attracted extensive interest for its potential in tackling crises of energy and environmental pollution. Apart from the two basic requirements for band gap and band-edge positions, (Fujishima and Honda, 1972), excellent light absorption, low carrier recombination, and considerable carrier mobility are also necessary for a superior photocatalyst (Li et al., 2016). Extensive experimental and theoretical studies have been conducted to explore efficient novel photocatalysts.

In recent years, emerging two-dimensional (2D) materials have opened up a colorful stage for the design of new photocatalysts (Fan et al., 2021; Ren et al., 2022a; Qin et al., 2022). Notably, the naturally high surface area of 2D materials can provide more active sites for catalytic reactions (Ganguly et al., 2019). Furthermore, 2D materials shorten the migration distance of photogenerated carriers, thereby reducing the recombination of an electron–hole pair (Fan et al., 2021). A large number of 2D materials have been developed for photocatalysis, such as transition-metal dichalcogenide (Voiry et al., 2016), MXene (Cheng et al., 2019), carbonitrides (Wang et al., 2009), and others (Roger et al., 2017). In 2015, Mohammed et al. synthesized a new 2D multifunctional material C_2_N with a band gap of 1.96eV (Mahmood et al., 2015). The C_2_N monolayer possesses phonon modes close to those of graphenes (Sahin, 2015), indicating its fine thermal stability. Many facts confirm the highly tunable photocatalytic ability of monolayered C_2_N for water splitting (Xu et al., 2015; Ashwin Kishore and Ravindran, 2017). However, the rapid recombination of photogenerated carriers is still a serious issue for the use of the C_2_N monolayer in water splitting (Xu et al., 2015).

Some engineering processes have been proposed to improve the photocatalytic performance of monolayer C_2_N for water splitting, including doping (Du et al., 2016), defects (Zhang et al., 2018), atomic adsorption (Kishore et al., 2019; Zhang et al., 2022), and strain (Guan et al., 2015). More recently, nascent van der Waals (vdW) heterostructures (Ye et al., 2019; Zhao et al., 2021; Wang et al., 2022) have also been widely considered as a means of promoting the photocatalytic water-splitting performance of the C_2_N monolayer. The vdW heterostructure, composed of different 2D components, can maintain the excellent properties of those components, while some novel properties may be generated due to the interlayer coupling effects (Novoselov et al., 2016). Notably, the electron–hole pair, separated on the constituent monolayers, can substantially reduce the recombination rate of carriers, which is indeed favorable for photocatalytic water splitting (Deng et al., 2016; Novoselov et al., 2016). Kumar found that the carrier mobilities of the C_2_N/WS_2_ heterostructure with photocatalytic potential are efficiently enhanced (Kumar et al., 2018). Theoretical studies reveal that the C_2_N/GaTe, C_2_N/InTe, and C_2_N/InSe heterostructures are all suitable for photocatalysis, while their photocatalytic properties are sensitive to strain (Wang et al., 2019; Wang X. et al., 2020). The vdW heterostructures, such as the C_2_N/Janus monochalcogenides (Ma et al., 2021), CdS/C_2_N (Luo et al., 2017), and h-BN/C_2_N (Wang G. et al., 2020), are also predicted to have excellent photocatalytic performance. Recently, the novel ZnO/C_2_N heterostructure with a direct band gap of 2.0 eV has been reported, and its optoelectronic properties can be tuned with vertical strain and an external electric field (Song et al., 2021). Monolayered ZnO with its graphene-like structure is a 2D photocatalytic material with high carrier mobility. However, its large band gap (∼3.3 eV) (Ren et al., 2020) leads to poor absorption, limiting its photocatalytic application. Perhaps the ZnO/C_2_N heterostructure formed by two photocatalysts has more prominent carrier mobility and photocatalytic performance, but this remains unknown thus far.

In this work, theoretical work is conducted to comprehensively explore the electronic structure, carrier mobility, hydrogen evolution reaction (HER), and absorption properties of the ZnO/C_2_N heterostructure, as well as the effect of lateral strain on these properties. All the results confirm the substantial application potential of a ZnO/C_2_N heterostructure in photocatalysis for water splitting.

## Computational methods

All calculations are implemented with the Vienna Ab-initio Simulation Package (VASP), based on the projected augmented wave method (PAW) (Kresse and Furthmüller, 1996a; Kresse and Furthmüller, 1996b). The generalized gradient approximation within the Perdew–Burke–Ernzerhof scheme (GGA-PBE) is used to describe the exchange-correlation functional (Perdew et al., 1996), while the DFT-D3 correction method (Grimme et al., 2010) is utilized to describe the vdW interaction between the two monolayers. The Heyd–Scuseria–Ernzerh hybrid functional (HSE06) (Heyd et al., 2003) is also adopted to determine the band gap of the ZnO/C_2_N heterostructure and its pristine components. The lattice constants and atomic positions of pristine ZnO and C_2_N monolayers are fully relaxed using the 6 × 6 × 1 and 15 × 15 × 1 *G*-centered Monkhorst–Pack (Monkhorst and Pack, 1976) *k*-mesh scheme to simplify the Brillouin zone, while a 3 × 3 × 1 *k*-mesh sampling is chosen for the ZnO/C_2_N heterostructure. All ion relaxation processes interrupt the process until the force per atom is less than 0.01 eV/Å and the energy convergence criterion of 10^–5^ eV is set. The plane wave cutoff of 450 eV is used throughout this work, and a 20 Å vacuum toward the *z*-direction is applied to shield interaction between neighboring layers. VASPKIT and VESTA are used for visualization (Momma and Izumi, 2011; Wang et al., 2021).

## Results and discussions

The geometric structures of ZnO and C_2_N monolayers are shown in Supplementary Figures S1A–C. Their lattice parameters are found to be 3.29 Å and 8.32 Å, respectively, which is consistent with previous reports (Mahmood et al., 2015; Lee et al., 2016). Both monolayers are direct band gap semiconductors, as the band structures are presented in Supplementary Figures S1B–D. The band gap values of ZnO and C_2_N determined by HSE06 are 3.28 eV and 2.47 eV, which are close to the reported results (Mahmood et al., 2015; Lee et al., 2016).

**FIGURE 1:**
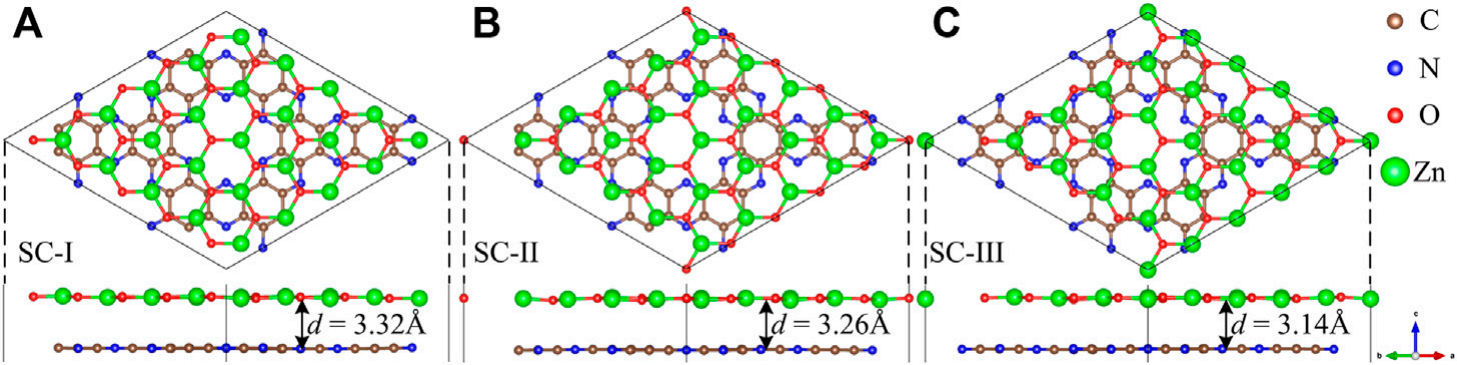
Optimized geometric structures of the **(A)** SC‐I, **(B)** SC‐II, **(C)** SC‐III ZnO/C_2_N heterostructure.

In order to minimize the strain effect, a 5 × 5 ZnO supercell and a 2 × 2 C_2_N supercell are used to make up the ZnO/C_2_N heterostructure. The ZnO/C_2_N heterostructure is constructed by fixing the C_2_N layer and shifting the ZnO layer to a high symmetry location. According to the location of the ZnO layer in the lattice, three kinds of stacking configurations (SCs) for the ZnO/C_2_N heterostructure are formed, as shown in Figure 1. In the interests of thermodynamic stability and for determining the most stable SC of the ZnO/C_2_N heterostructure, the binding energy *E*_b_ and the formation energy *E*_f_ are calculated using the Supplementary Equations S1, S2. According to the results in Supplementary Table S1, *E*_f_ and *E*_b_ are close to those of typical vdW heterostructures (Guo et al., 2017; Fan et al., 2019; Bafekry et al., 2020; Guo et al., 2020). The negative values confirm that all ZnO/C_2_N heterostructures can be prepared experimentally, as their stabilities are slightly different. The ZnO/C_2_N heterostructure in SC-Ⅲ, with an interlayer distance *d* of 3.14 Å, is provided with the most beneficial stability. Therefore, the ZnO/C_2_N heterostructure in SC-Ⅲ is the focus of the following research. Moreover, the *ab initio* molecular dynamic (AIMD) simulation is performed at 300 K to check the thermodynamic stability of the ZnO/C_2_N heterostructure. As the snapshot for the last frame shows in Supplementary Figure S3A, the ZnO/C_2_N heterostructure maintains good structural integrity within 6 ps, demonstrating its stability at room temperature. The time-dependent evolution of total potential energies, exhibited in Supplementary Figure S3B, also proves its thermal stability.

**FIGURE 2:**
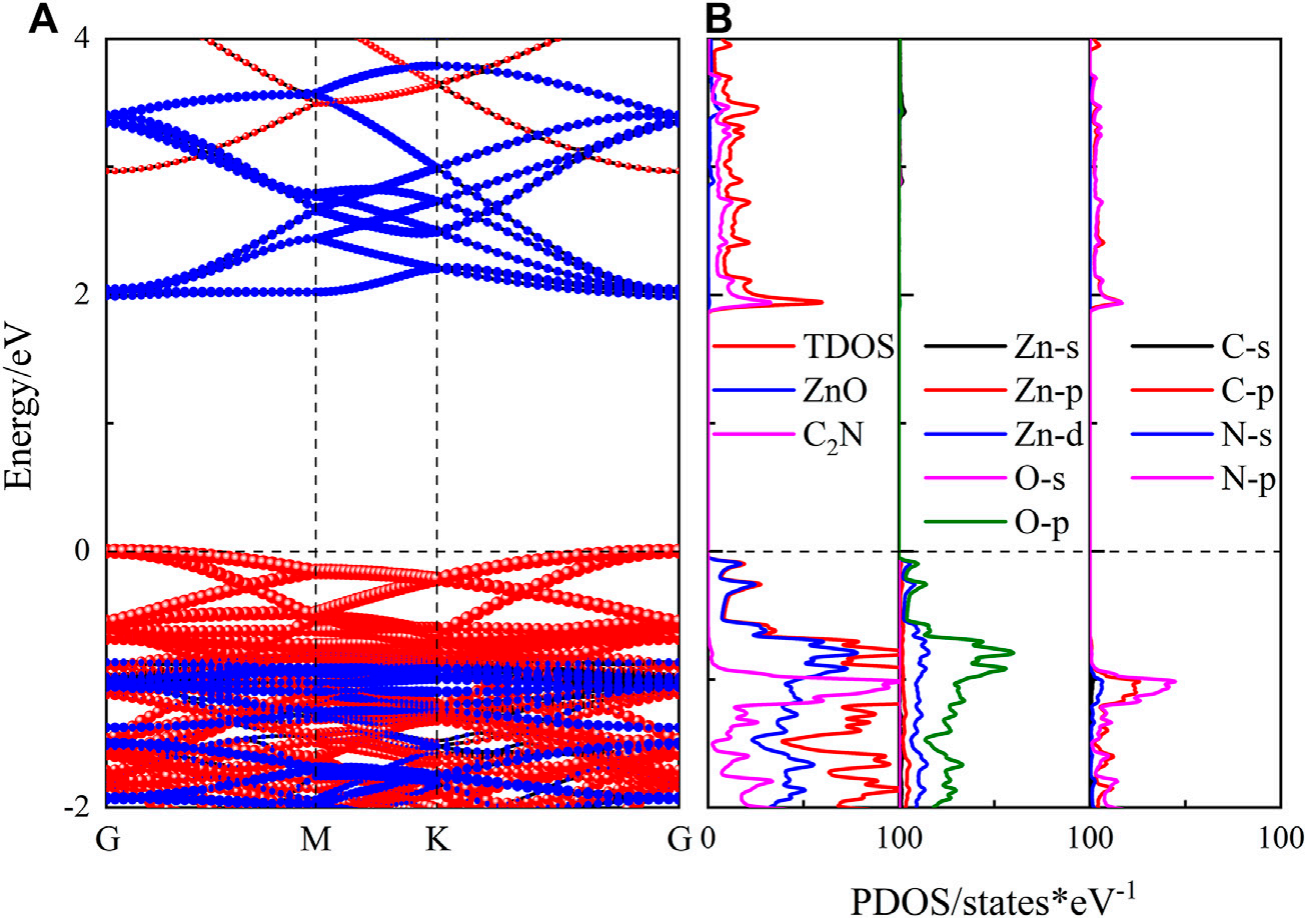
**(A)** Projected band structure and **(B)** PDOS of the ZnO/C_2_N heterostructure.

The band structures of the ZnO/C_2_N heterostructure within the three SCs are calculated, together with the projected density of states (PDOS). As shown in Figure 2A, the ZnO/C_2_N heterostructure is a direct band gap semiconductor, as both the VBM and CBM emerge at the *G*-point. Results of band structures also indicate that the SC has a negligible impact on electronic property. ZnO/C_2_N heterostructures in three SCs possess the same band gap of 0.77 eV and 1.99 eV, based on the PBE functional and the HSE06 functional, respectively. The band gaps are smaller than those of pristine monolayers, meaning that forming a heterostructure can evidently reduce the band gap and widen the range of absorption. Moreover, both the Figures 2A,B demonstrate that a type‐II heterostructure is formed when ZnO comes into contact with C_2_N.

**FIGURE 3:**
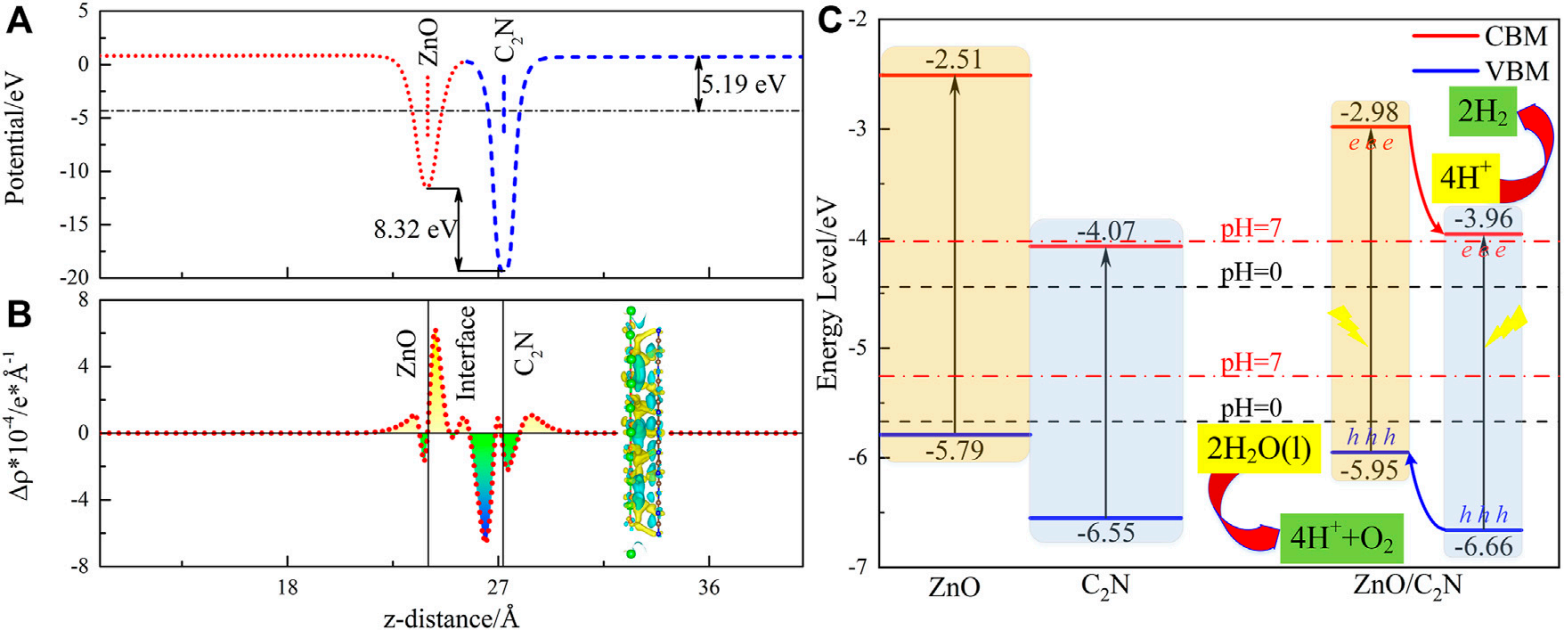
**(A)** Electrostatic potential, **(B)** planar-average and visual charge density difference Δ*ρ*, and **(C)** band alignment for the ZnO/C_2_N heterostructure.

Figure 3 shows the electron transfer between two component layers. Work function (*W*_f_) is a serious parameter defining the ability of a catalyst surface to attract electrons (Vayenas et al., 1990). The values of *W*_f_ for ZnO and C_2_N are calculated as 4.82 eV and 5.78 eV, portending that electron flow from the ZnO layer to the C_2_N layer at the interface. Electron migration from ZnO to C_2_N is also observed from the planar-averaged charge density difference Δ*ρ* in Figure 3B, which ceases until the Fermi level is aligned, producing the positively charged ZnO layer and the negatively charged C_2_N layer. The visual charge density difference is also exhibited in Figure 3B, in which the yellow and cyan marked areas represent electron accumulation and electron depletion, respectively. Therefore, a potential drop of 8.32 eV is formed in Figure 3A. Finally, a built-in electric field, pointing from ZnO to C_2_N, is generated at the interface. The Bader charge calculation (Tang et al., 2009) also demonstrates the result of 0.203 electrons transferred from ZnO to C_2_N. It is worth noting that the prominent potential drop in the ZnO/C_2_N heterostructure can also offer a critical promotion for the separation of the photogenerated electron and hole, thereby reducing the recombination of carriers.

Suitable band edge positions (*E*_VBM_ and *E*_CBM_) and decent band alignment are crucial for photocatalysis (Zheng et al., 2018; Tang et al., 2020). The method proposed by Toroker et al. (2011) has been employed to evaluate the *E*_VBM_ and *E*_CBM_ of the ZnO/C_2_N heterostructure so as to explore its potential as a photocatalyst. It is obvious from Figure 3C that both the *E*_VBM_ and *E*_CBM_ of the two monolayers (Wang et al., 2018; Zhang X. et al., 2019) and the ZnO/C_2_N heterostructure, meet the redox potential requirements for a photocatalyst in an acidic environment (pH = 0). The ZnO/C_2_N heterostructure still has the talent of photocatalysis for water splitting in a neutral environment with pH = 7. Furthermore, a higher CBM and VBM of ZnO than those of C_2_N can be observed. It is thus suggested that hydrogen evolution reaction (HER) occurs on the C_2_N layer, while an oxygen evolution reaction (OER) happens on the ZnO layer. We then expand the type-Ⅱ mechanism in the ZnO/C_2_N heterostructure to boost HER and OER for water splitting. The conduction band offset (CBO) and valence band offset (VBO) are calculated as 0.98 eV and 0.69 eV, respectively. When the heterostructure is irradiated, the CBO promotes the transfer of photogenerated electrons in the CB of the ZnO layer to the CB of the C_2_N layer. The photogenerated holes in the C_2_N layer are driven by the VBO to the VB of the ZnO layer. Finally, the photogenerated electrons and holes remain in the C_2_N and ZnO monolayers, respectively, bringing about carrier separation spatially. Naturally, the type-II band alignment of the ZnO/C_2_N heterostructure is instrumental in overcoming the recombination of carriers to achieve better photocatalytic performance.

High carrier mobility *μ* is essential for superior photocatalysts (Guo et al., 2020; Ren et al., 2022b). The carrier mobility *μ*, defined as

$$\mu =\frac{2e\hbar^{3}C_{2D}}{3k_{B}T{m^{*}}^{2}E_{1}^{2}},$$ (1)

has been evaluated based on the deformation potential theory (Bardeen and Shockley, 1950) for both the ZnO/C_2_N heterostructure and the two monolayers. The methods and details are mentioned in Supplementary Section S1. As for the results of the ZnO monolayer listed in Table 1, the electron mobilities in the *x*- and *y*-directions are superior to those of the hole, conforming to previous theoretical (Ren et al., 2020) and experimental (Gonzalez-Valls and Lira-Cantu, 2009; Anta et al., 2012) results. It can be observed from Table 1 that the excellent electron mobilities of the C_2_N monolayer are 4265 and 1944 cm^2^/V/s in the *x*- and *y*-directions, mainly due to the small carrier effective mass (*m* ^*^) and deformation potential constant (*E*_1_). However, the hole *m* ^*^ of the C_2_N monolayer is several times that of the electron, resulting in low hole mobility (Kumar et al., 2018; Zhang X. et al., 2019).

**TABLE 1:** Carrier mobilities of the ZnO/C_2_N heterostructure and two monolayers.

Carrier	System	*m* ^*^_x_ (*m*_0_)	*m* ^*^_y_ (*m*_0_)	*E*_1x_ (eV)	*E*_1y_ (eV)	*C*_2D_x_ (N/m)	*C*_2D_y_ (N/m)	*μ*_x_ (cm^2^/V/s)	*μ*_y_ (cm^2^/V/s)
*e*	ZnO	0.21	0.25	5.91	5.38	51.76	51.65	477.8	406.1
	C_2_N	0.46	0.42	1.59	2.58	160.45	160.57	4265.2	1944.5
	ZnO/C_2_N	0.14	0.14	7.36	5.85	205.78	205.66	2756.0	4359.9
*h*	ZnO	0.58	0.49	5.42	5.15	51.76	51.65	74.5	115.3
	C_2_N	10.64	6.05	3.41	3.29	160.45	160.57	1.7	5.7
	ZnO/C_2_N	0.62	0.60	3.79	4.53	205.78	205.66	529.9	395.9

The hole *m* ^*^s of the ZnO/C_2_N heterostructure in the *x-* and *y*-directions are close to those of the ZnO monolayer, while the values of *E*_1_ in the aforementioned directions are comparable to those of the C_2_N monolayer. The in-plane stiffness (*C*_2D_) of the ZnO/C_2_N heterostructure increases and is about equal to the sum of two component layers. Consequently, the hole mobilities of the heterostructure in the *x*- and *y*-directions are 529.9 and 395.9 cm^2^/V/s, respectively, where pronounced improvements are due to the abovementioned changes of *m* ^*^, *E*_1_, and *C*_2D_. It can be deduced that higher carrier mobility will induce enhanced carrier separation and migration in the ZnO/C_2_N heterostructure, which should illuminate its photocatalytic prospects for application in water splitting.

In order to explore the kinetic behavior of water splitting, the Gibbs free energy difference Δ*G* of HER and OER on the ZnO/C_2_N heterostructure is calculated using the method developed by Nørskov et al. (2005). The calculation details and favor absorption sites are present in the Supplementary Material. The HER is divided into the following two reactions:

$${}^{*}+H^{+}+e^{-}\to H^{*},$$ (2)

$$H^{*}+H^{+}+e^{-}\to {H_{2}+}^{*}.$$ (3)

**FIGURE 4:**
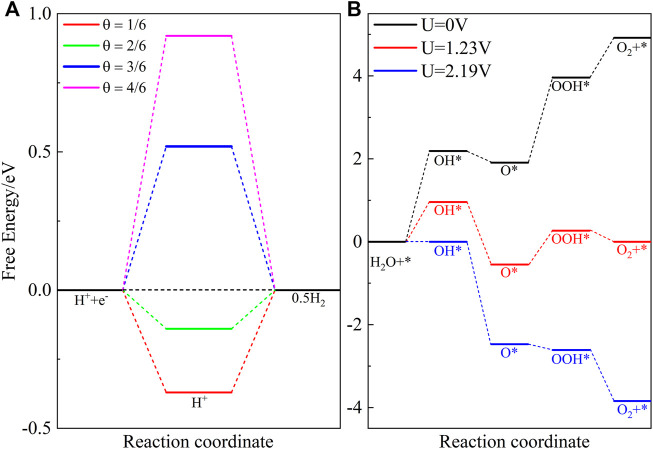
Free energy differences of **(A)** HER and **(B)** OER steps.

H^∗^ is the only intermediate of HER, and it is obvious in Figure 4A that Δ*G* is a function of H coverage *θ*. When the *θ* equals 2/6, Δ*G* can be as low as 0.14 eV, which is comparable to the value of the C_2_N/WS_2_ heterostructure (Kumar et al., 2018). Therefore, the ZnO/C_2_N heterostructure can be used as a potential photocatalyst for HER due to the small value of Δ*G* (Hinnemann et al., 2005).

The OER involves the following four steps:

$${}^{*}+H_{2}O\to {OH}^{*}+H^{+}+e^{-},$$ (4)

$${OH}^{*}\to O^{*}+H++e^{-},$$ (5)

$$O^{*}+H_{2}O\to {OOH}^{*}+H++e^{-},$$ (6)

$${OOH}^{*}\to *+O_{2}+H++e^{-}.$$ (7)

Figure 4B shows the first step, with an overpotential of 2.19 V, is the limiting step when no external potential is applied. Under the action of 1.23 V external potential, the overpotential reduces to 0.96 V. As the value of the extra potential increases to 2.19 V, all the OER steps are downhill, suggesting that these reaction steps are exothermic.

The performance of absorption is an important function of a photocatalyst, as it is the first step in water splitting to produce electron–hole pairs. Superior absorption with a wide range and a high coefficient is essential for a photocatalyst’s effective solar energy utilization. The optical coefficients *α*(ω) of the ZnO/C_2_N heterostructure and two components are calculated with the following equation (Gajdoš et al., 2006):

$$\alpha \left(\omega \right)=\sqrt{2}\omega {\left[\sqrt{\varepsilon_{1}{\left(\omega \right)}^{2}+\varepsilon_{2}{\left(\omega \right)}^{2}}-\varepsilon_{1}\left(\omega \right)\right]}^{1/2}.$$ (8)

**FIGURE 5:**
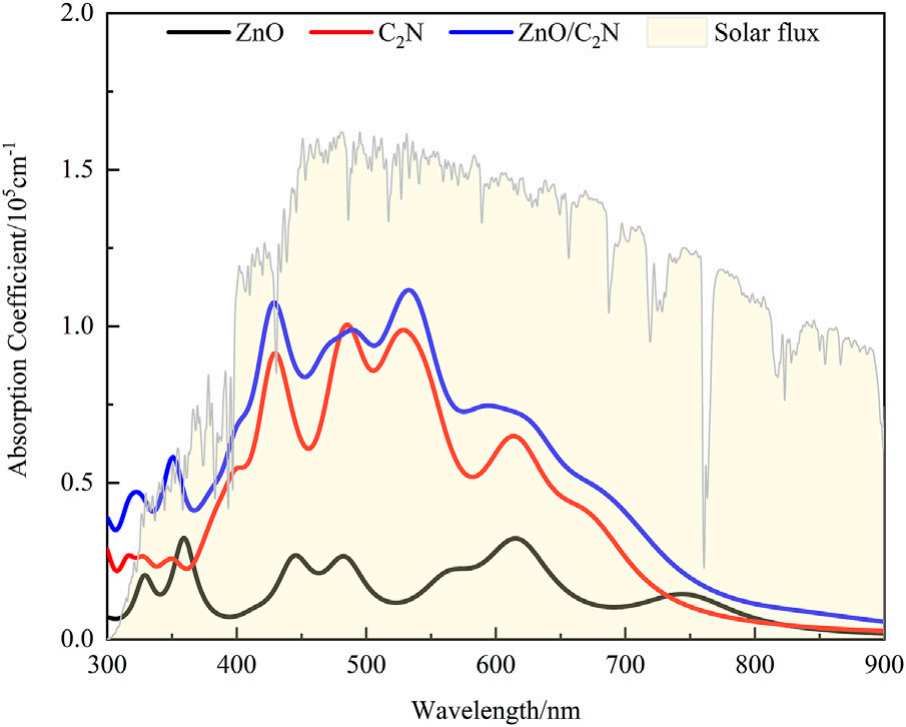
Absorption spectrum of the ZnO/C_2_N heterostructure compared with ZnO and C_2_N.

In this equation, *ω*_1_ and *ω*_2_ represent the real and imaginary parts of the dielectric function, respectively. The result, displayed in Figure 5, demonstrates the advantage of the heterostructure in absorption over ZnO and C_2_N. The heterostructure not only possesses a higher absorption coefficient (∼10^5^cm^−1^) than ZnO and C_2_N but also has a wider absorption range from visible to ultraviolet light. The improvement in absorption performance can be attributed to its reduced band gap and significantly improved carrier mobility. The excellent absorption ability can generate more electron–hole pairs in the first step of water splitting, which is beneficial for the ZnO/C_2_N heterostructure in realizing its efficient photocatalytic performance.

**FIGURE 6:**
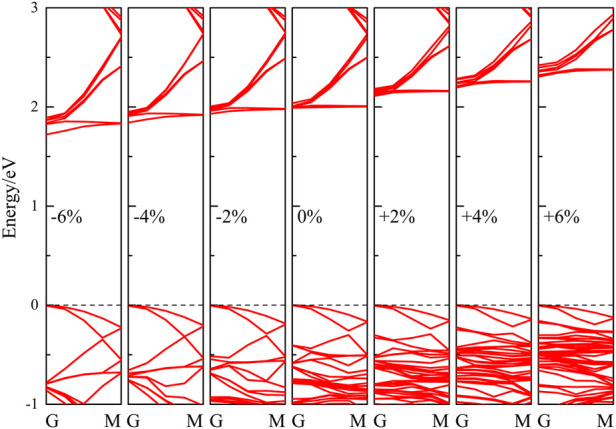
Calculated band structures of strained ZnO/C_2_N heterostructures.

The lateral strain is a common effect in heterostructures, as well as being a proven effective means of improving the photocatalytic performance of 2D material (Feng et al., 2012; Zhang J.-R. et al., 2019). We thus undertook a full investigation of the electronic and optical properties of the ZnO/C_2_N heterostructure with biaxial lateral strain to explore the regulatory effect of strain on photocatalytic performance. Strain ranging from −6% to +6% was applied to the ZnO/C_2_N heterostructure, and the band structures calculated with the HSE06 functional in Figure 6 clearly announce the structure’s identity as a direct band gap semiconductor.

**FIGURE 7:**
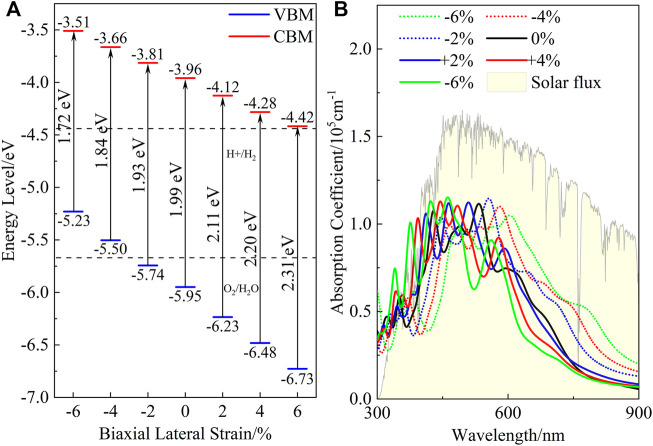
**(A)** Band alignment and **(B)** absorption spectrum of ZnO/C_2_N heterostructure under different strain conditions.

Meanwhile, it can be seen from the PDOS exhibited in Supplementary Figure S13 that the strained heterostructures also belong to type-Ⅱ heterostructures, as both the VBM and CBM of the ZnO are higher than those of C_2_N. Moreover, it is clear in Figure 7A that compressive strain reduces the band gap, while tensile strain increases the gap. When the lattice is compressed by 6%, the band gap decreases to 1.72 eV, while the gap value increases to 2.31 eV when the heterostructure it is expanded by 6%. Within the strain range of −2% to +6%, we can also see that the band edge positions of the heterostructure still meet the requirements of photocatalysis for water splitting at the condition of pH = 0. It is very important for heterostructures to maintain their photocatalytic ability across a wide pH range (Ren et al., 2019). The band alignment shown in Supplementary Figure S15 indicates that the strained heterostructures still possess potential application for water splitting across a wide pH range. Figure 7B shows the effect of the strain on the absorption performance of the ZnO/C_2_N heterostructure. Compared with the freestanding ZnO/C_2_N heterostructure, the compressed heterostructures have higher absorption intensity and a wider absorption range, while the lattice expansion leads to improvement in the absorption performance of the heterostructures in the ultraviolet range. The significant modification of absorption is mainly a benefit of the regulation of the band gap. All the results directly confirm that the ZnO/C_2_N heterostructure is a promising candidate for use in the field of water splitting.

## Conclusion

In this study, the electronic and absorption properties of the ZnO/C_2_N heterostructure are explored to reveal its potential for water splitting. The stabilized heterostructure is given a reduced band gap of 1.99 eV, while its band edge positions also meet the water-splitting requirements. The band alignment of the heterostructure belongs to type-II, which leads to the generation of a built-in electrical field between the two layers that promote carrier separation and migration. The more significant change is that the carrier mobility of the ZnO/C_2_N heterostructure is several times improved. The results of the Gibbs free energy calculation clearly indicate the promising catalytic ability of the ZnO/C_2_N heterostructure. As for the optical absorption performance, the reduced band gap and excellent carrier mobility endow the ZnO/C_2_N heterostructure with considerable absorption intensity and a wider absorption range. Moreover, the electronic and absorption properties of the ZnO/C_2_N heterostructure can be substantially tuned with biaxial lateral strain. All the results confirm that the ZnO/C_2_N heterostructure has potential use as a superior photocatalyst for water splitting.

## Data Availability

The original contributions presented in the study are included in the article/Supplementary Material, and further inquiries can be directed to the corresponding author.
