# Supplementary material for: Theoretical study on photocatalytic performance of ZnO/C2N heterostructure towards high efficiency water splitting
Source: Front Chem. 2022 Oct 19;10:1048437. doi: 10.3389/fchem.2022.1048437 (PMC9626801; doi:10.3389/fchem.2022.1048437)
Supplement: Supplementary file 1 [file DataSheet1.docx]

**Theoretical study on photocatalytic performance of ZnO/C_2_N heterostructure towards high efficiency water splitting**

Meiping Liu^1,3^, Yong Tang^2,3*^, Haizi Yao^2^, Liuyang Bai^2^, Jun Song^2^, Benyuan Ma^2^

1. Henan Key Laboratory of Smart Lighting, Huanghuai University, Zhumadian, 463000, Henan, China;
2. School of Energy Engineering, Huanghuai University, Zhumadian, Henan 463600, China;
3. School of Materials Science and Engineering, Xiangtan University, Xiangtan, Hunan, 411105, China.

*Corresponding E-mail: 20202127@huanghuai.edu.cn

**Section 1: Detailed computing methods of** **binding energy *E*_b_, formation energy *E*_f_, and carrier mobility *µ***

When ZnO layer is moved into the lattice of the heterostructure, the heterostructure is named as SC-Ⅰ. If the Oxygen atoms or Zinc atoms are located at the edge of the lattice, the heterostructure is named SC-Ⅱ or SC-Ⅲ, respectively

1. The **binding energy *E*_b_** and **formation energy *E*_f_** are determined by:

$E_{b}=\frac{E_{het}-E_{ZnO}-E_{C2N}}{S_{0}}$…………………………………....……………...……..…......(S1)

$E_{f}=\frac{E_{het}-{N_{Zn}*E}_{Zn}{-N_{O}*E}_{O}-{N_{C}*E}_{C}-{N_{N}*E}_{N}}{S_{0}}$…………………………………….……....(S2)

In these equations, *E*_het_ and *S*_0_ represent the total energy and the surface area of ZnO/C_2_N heterostructures. *E*_ZnO_ and *E*_C2N_ are the energies of ZnO and C_2_N layer, respectively. *E*_Zn_, *E*_O_, *E*_C_, and *E*_N_ express the average energy of a single atom in its most stable phase, respectively. Correspondingly, the coefficients of *N*_Zn_, *N*_O_, *N*_C_, and *N*_N_ are defined as the number of various atoms in the ZnO/C_2_N heterostructure.

1. The **carrier mobility *μ***, defined as(Bardeen and Shockley, 1950)：

$\mu_{2D}=\frac{2e\hbar^{3}C_{2D}}{3k_{B}T\left| m^{*} \right|^{2}E_{1}^{2}}$………………………………....……………………………....(S3)

In the S3 equation, *e*, *ħ*, *k*_B_, and *T* mean the electron charge, reduced Planck constant, Boltzmann constant, temperature, respectively. *T* = 300 K has been selected in this work. The carrier effective mass *m*^*^ is defined as *m*^*^ = *ħ^2^*(∂^2^*E_k_*/∂*k*^2^)^−1^. The in-plane stiffness *C*_2D_ is derived from *C*_2D_ = *S*_0_^−1^(∂^2^*E*/∂*δ*^2^), as *E*, *S*_0,_ and *δ* are the total energy of 2D systems, surface area, and strain, respectively. The deformation potential constant *E*_1_, referring to energy shift ∂*E_edg_* of VBM and CBM caused by strain *δ*, is expressed as *E*_1_ = ∂*E_edg_*/∂*δ*.

1. The **Gibbs free energy difference Δ*G*** is calculated with the following equation (Nørskov et al., 2005; Skúlason et al., 2010):

Δ*G* = Δ*E +* Δ*E*_ZPE_*-* *T*Δ*S +* Δ*G*_U_ *+* Δ*G*_pH_…………………………....……………...(S4)

in which, Δ*E*, Δ*E*_ZPE_ and *T*Δ*S* express the changes in total energy, zero-point energy and entropy, respectively. Δ*G*_U_ = -*eU*, in which U means the potential difference from the standard hydrogen electrode potential. Δ*G*_pH_ denotes the contribution of pH value on the free energy difference. Δ*G*_pH_ = 2.303*k*_B_*T* × pH, as *k*_B_ and *T* are the Boltzmann constant and reaction temperature. In this work, *T* and pH are set to 298.15K and 0, respectively. Therefore, the difference of free energy for HER is valued by:

Δ*G*_H_ = *G*_*H_ *-*1/2*G*_H_ *- G*_*_ *- eU -* Δ*G*_pH_…………………………....………………....(S5)

And while the free energy differences for OER with four steps are individually computed by:

Δ*G*_1_ = *G*_*OH_ + 1/2*G*_H2_ - *G*_*_ - *G*_H2O_ - *eU* + Δ*G*_pH_…………………………....……....(S6)

Δ*G*_2_ = *G*_*O_ + 1/2*G*_H2_ - *G*_*OH_ - *eU* + Δ*G*_pH_…………………………....…………....(S7)

Δ*G*_3_ = *G*_*OOH_ + 1/2*G*_H2_ - *G*_*O_ - *G*_H2O_ - *eU* + Δ*G*_pH_…………………………....…....(S8)

Δ*G*_4_ = *G*_*O2_ + 1/2*G*_H2_ + *G*_*_ - *G*_*OOH_ - *eU* + Δ*G*_pH_………………………………....(S9)

**Section 2: Supporting Tables and Figures**

**TABLE S1 |** The lattice constant *a*, interlayer distance *d*, formation energy *E*_f_, binding energy *E*_b_, band gap *E*_g_ and work function *W*_f_ of ZnO/C_2_N heterostructures.

| SC | *a*/Å | *d*/Å | *E*_f_/meV*Å^-2^ | *E*_b_/meV*Å^-2^ | *E*_g_/eV | *W*_f_/eV |
| --- | --- | --- | --- | --- | --- | --- |
| Ⅰ | 16.56 | 3.32 | -16.14 | -17.78 | 1.99(0.77) | 5.17 |
| Ⅱ | 16.56 | 3.26 | -16.18 | -17.80 | 1.99(0.77) | 5.18 |
| Ⅲ | 16.57 | 3.14 | -16.27 | -17.89 | 1.99(0.77) | 5.19 |

**TABLE S2 |** Values used for the entropy (T×S) and zero-point energy (ZPE) corrections in calculating the free energy of HER and OER.

| system | T×S(eV) | ZPE(eV) |
| --- | --- | --- |
| θ=1/6 | 0.01 | 1.27 |
| θ=2/6 | 0.03 | 2.51 |
| θ=3/6 | -0.01 | 3.73 |
| θ=4/6 | -0.02 | 5.05 |
| H_2_O(0.035bar) | 0.68 | 0.55 |
| H_2_ | 0.40 | 0.28 |
| *O | 0.04 | 0.32 |
| *OH | 0.21 | 0.33 |
| *OOH | 0.29 | 0.40 |


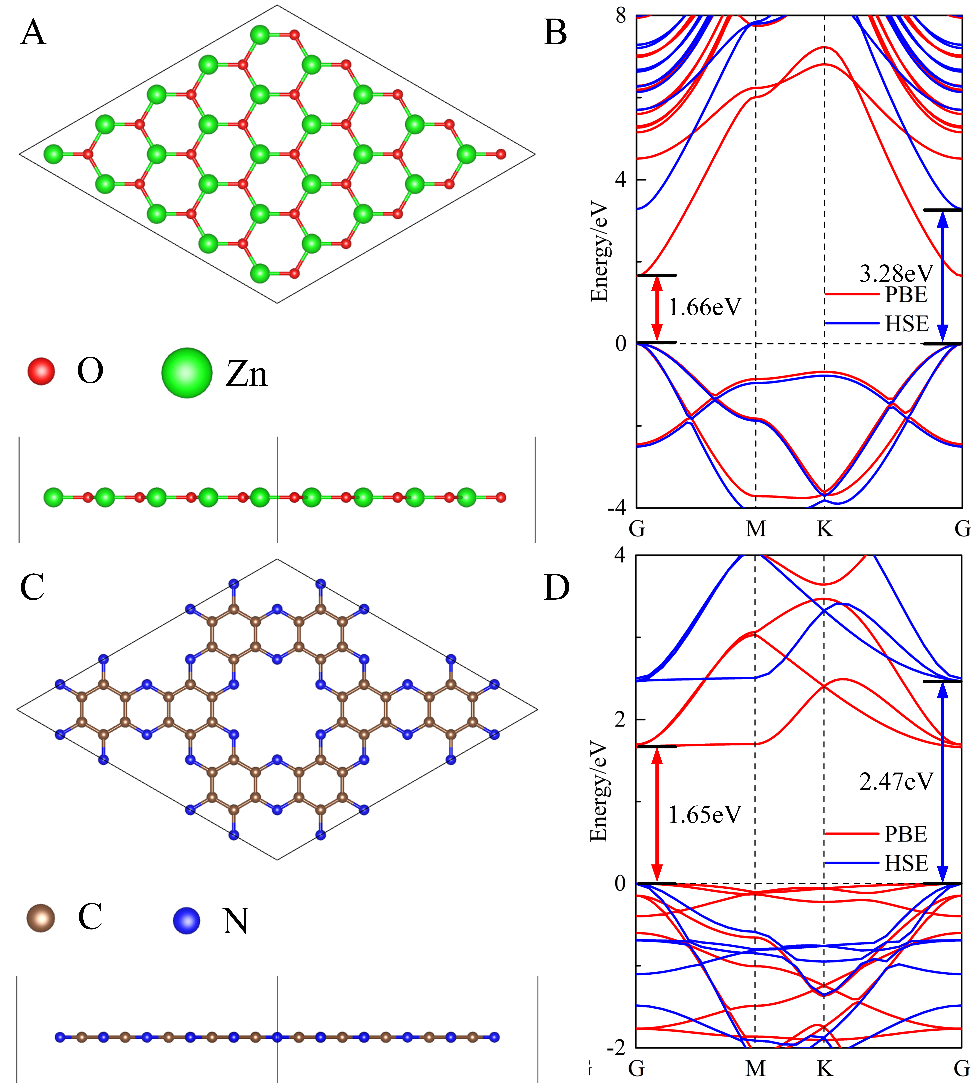


**Supplementary Figure S1**. Geometric structures of monolayered (A) ZnO and (C) C_2_N. Band structures of (B) ZnO and (D) C_2_N monolayers, with Fermi level locating at zero. The red and blue lines represent the results of PBE and HSE06 functionals, respectively.

The AIMD simulation is performed with Nosé-Hoover heat bath schemes in NVT ensemble, and it lasted for 6 ps with a time step of 1.0 fs. The temperature is set to 300K. Since the ZnO/C_2_N heterostructure cell contains 122 atoms, our computing resources are limited to support larger supercell. Therefore, AIMD simulation is carried out on its primitive cell, whose size is big enough to ensure the accuracy of the result.


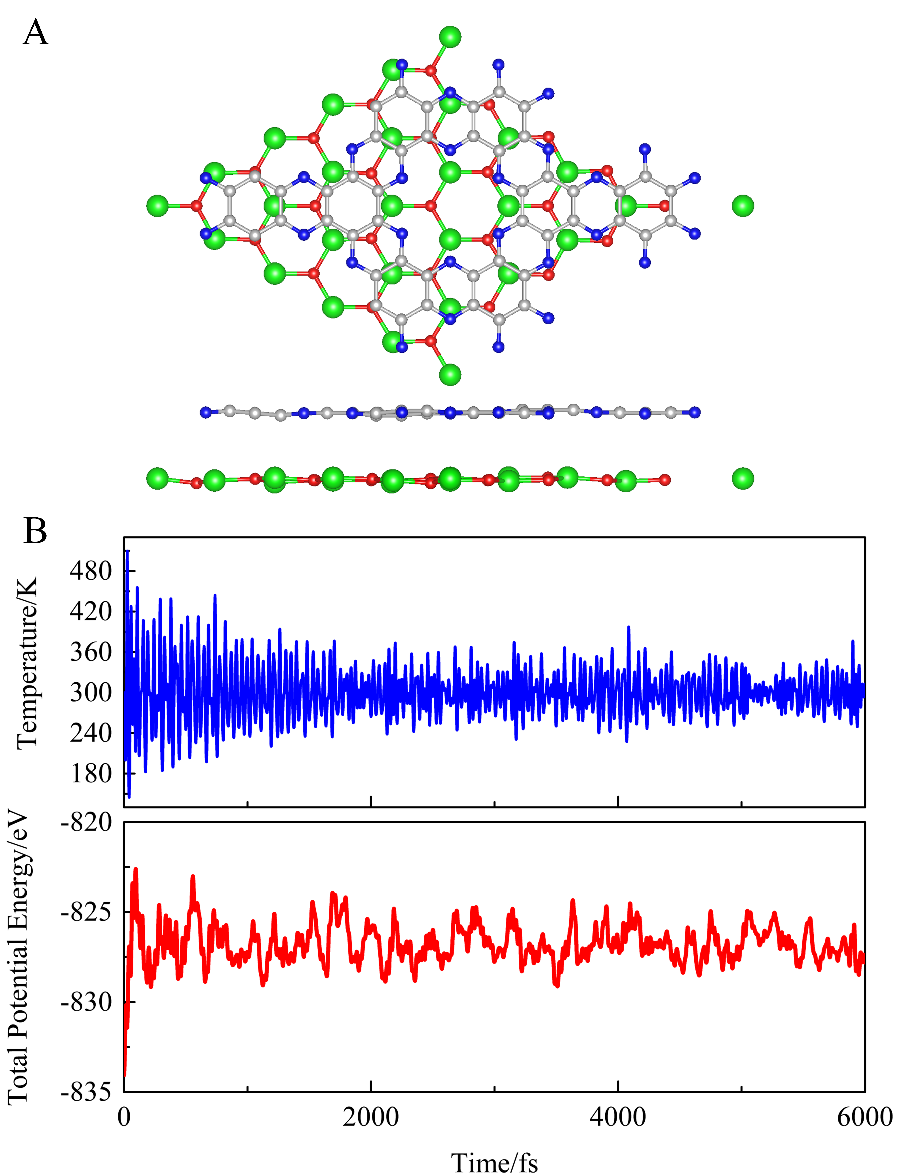


**Supplementary Figure S3**. (A) Structure snapshot of ZnO/C_2_N heterostructure at the last frame. (B) Temperature fluctuation and total potential energy during AIMD at 300 K of ZnO/C_2_N heterostructure.





**Supplementary Figure S4**. The projected band structures based on the HSE06 functional for the ZnO/C_2_N heterostructure in (a) SC-Ⅰ and (b) SC-Ⅱ.





**Supplementary Figure S5**. Calculated band structures based on the PBE functional for the ZnO/C_2_N heterostructures in SC-Ⅰ, SC-Ⅱ, and SC-Ⅲ.





**Supplementary Figure S6**. The projected band structures of each element for the ZnO/C_2_N heterostructure in SC-Ⅲ.


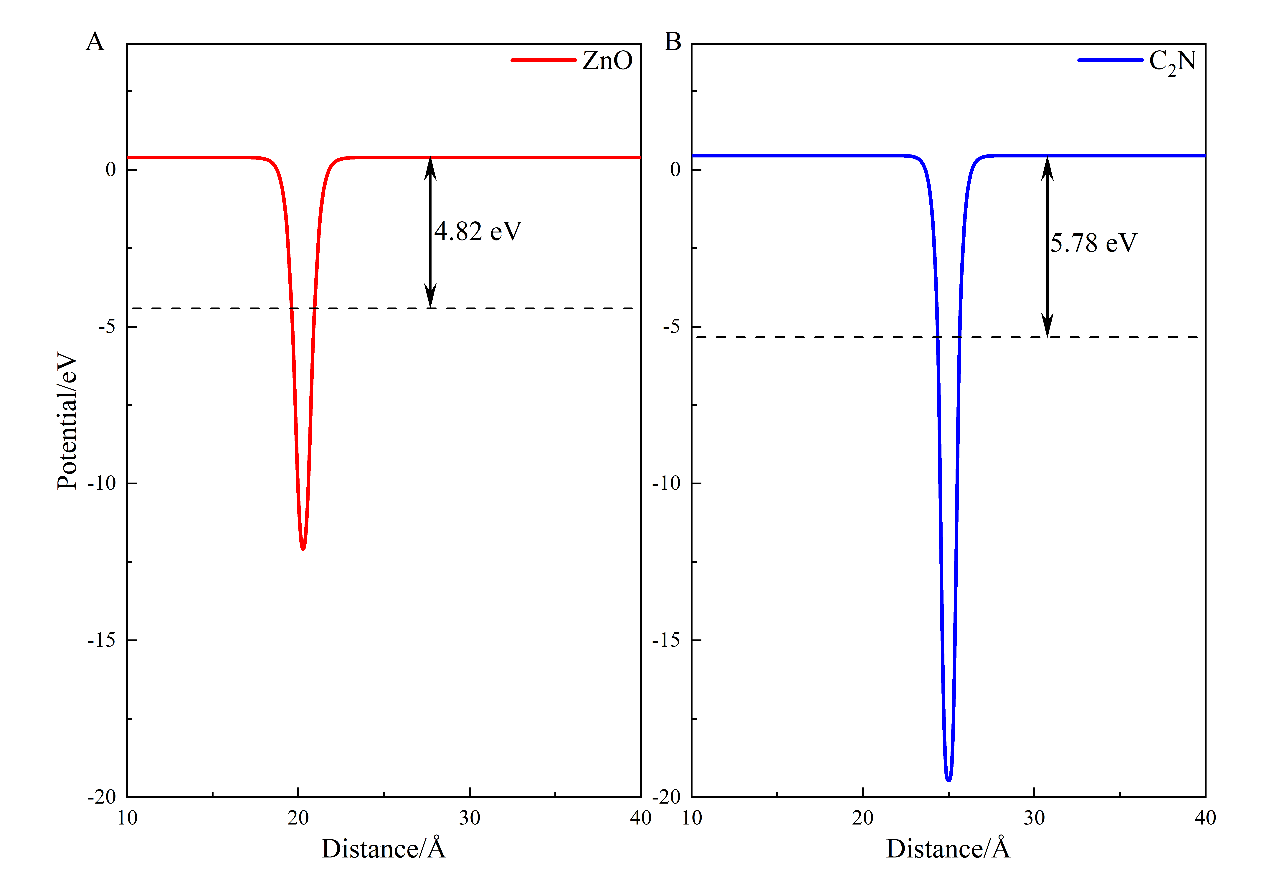


**Supplementary Figure S7**. The potential of monolayered (A)ZnO and (B)C_2_N before contact.


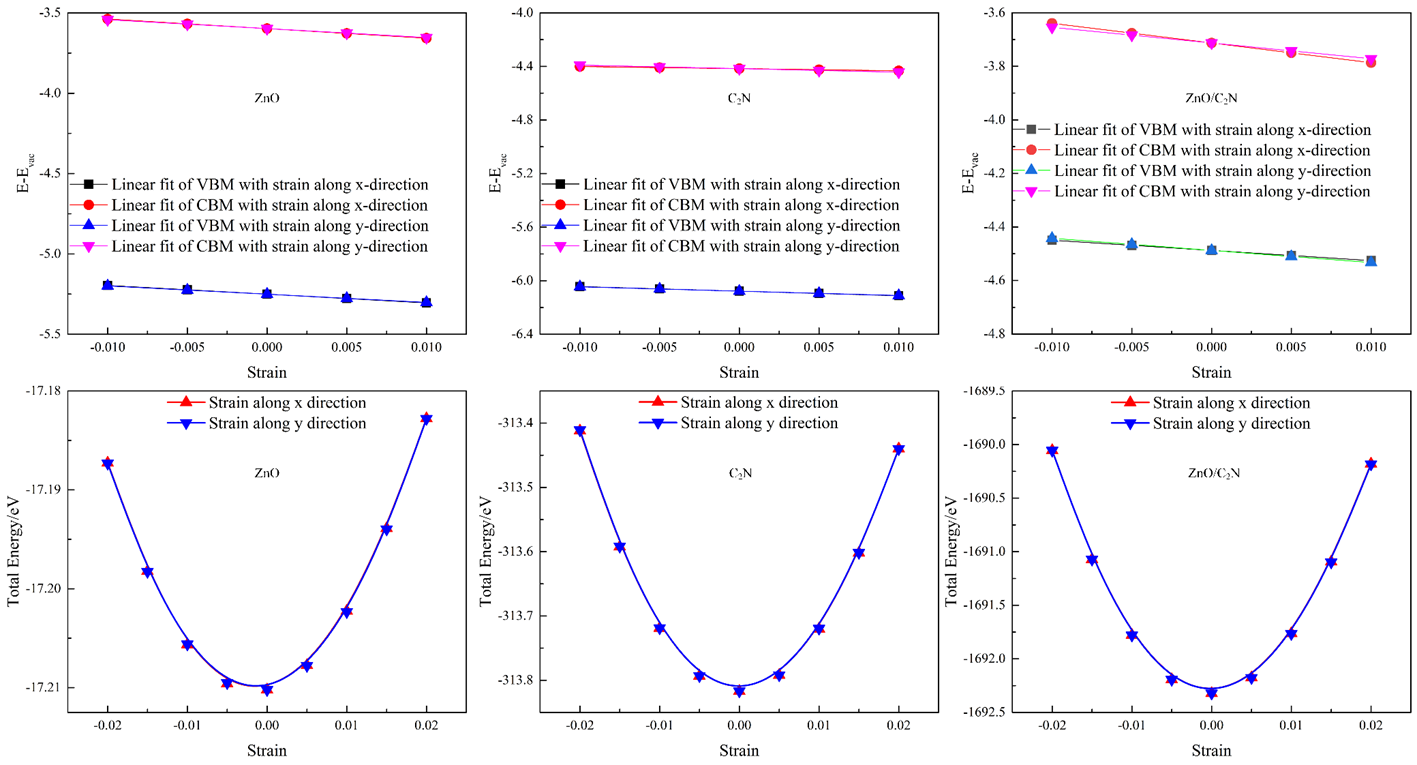


**Supplementary Figure S8**. The total energy-strain curve, and band edge position-strain curve of ZnO, C_2_N, and ZnO/C_2_N heterostructure in an orthorhombic cell.


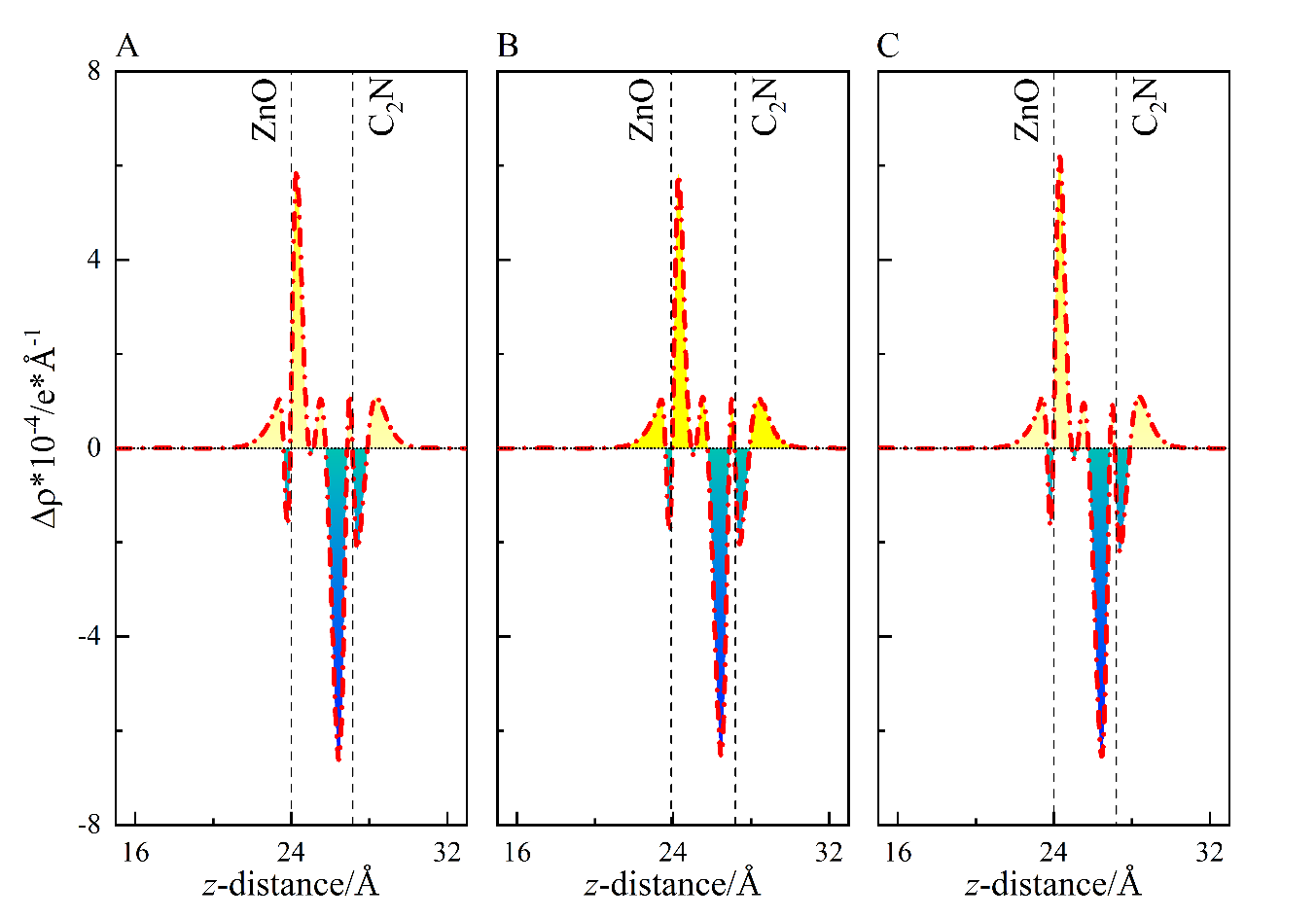


**Supplementary Figure S9**. The planar-average charge density difference Δ*ρ* for ZnO/C_2_N within (A) SC-Ⅰ, (B) SC-Ⅱ and (C) SC-Ⅲ.





**Supplementary Figure S10**. Absorption performances for the ZnO/C_2_N heterostructures in SC-Ⅰ, SC-Ⅱ, and SC-Ⅲ, compared with the pristine monolayers.


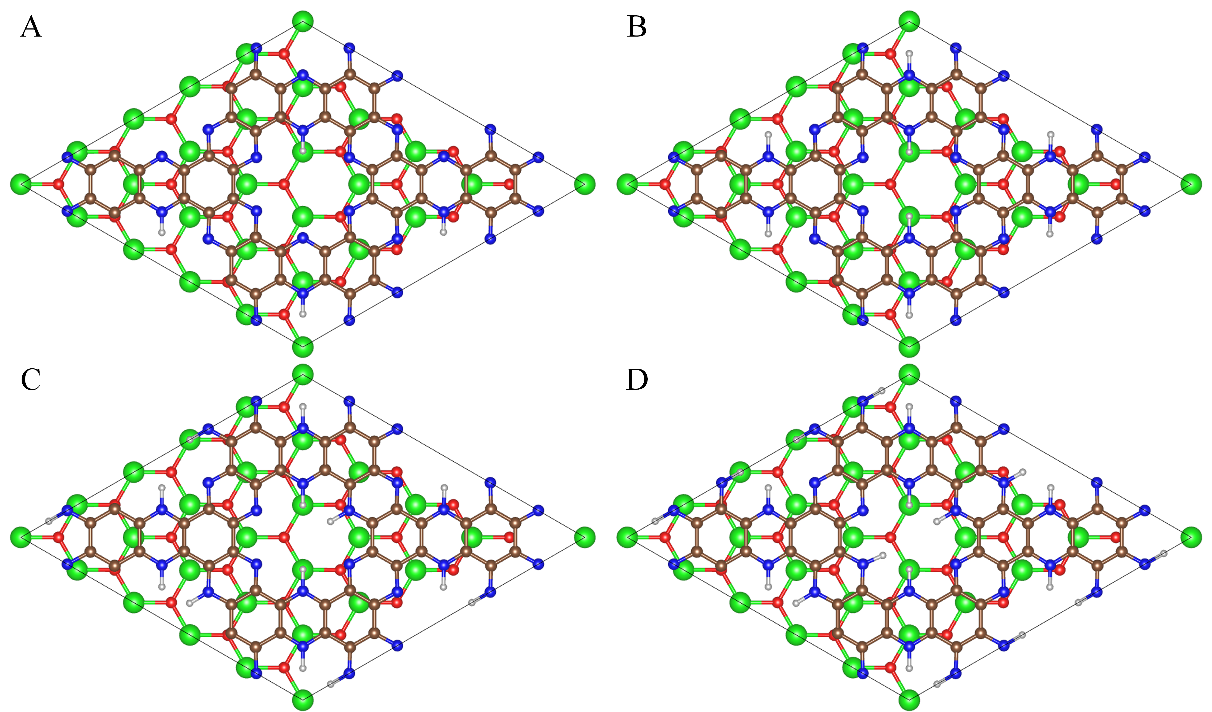


**Supplementary Figure S11**. The optimized geometries of (A) *-4H, (B) *-8H, (C) *-12H, (D) *-16H intermediates from top view.


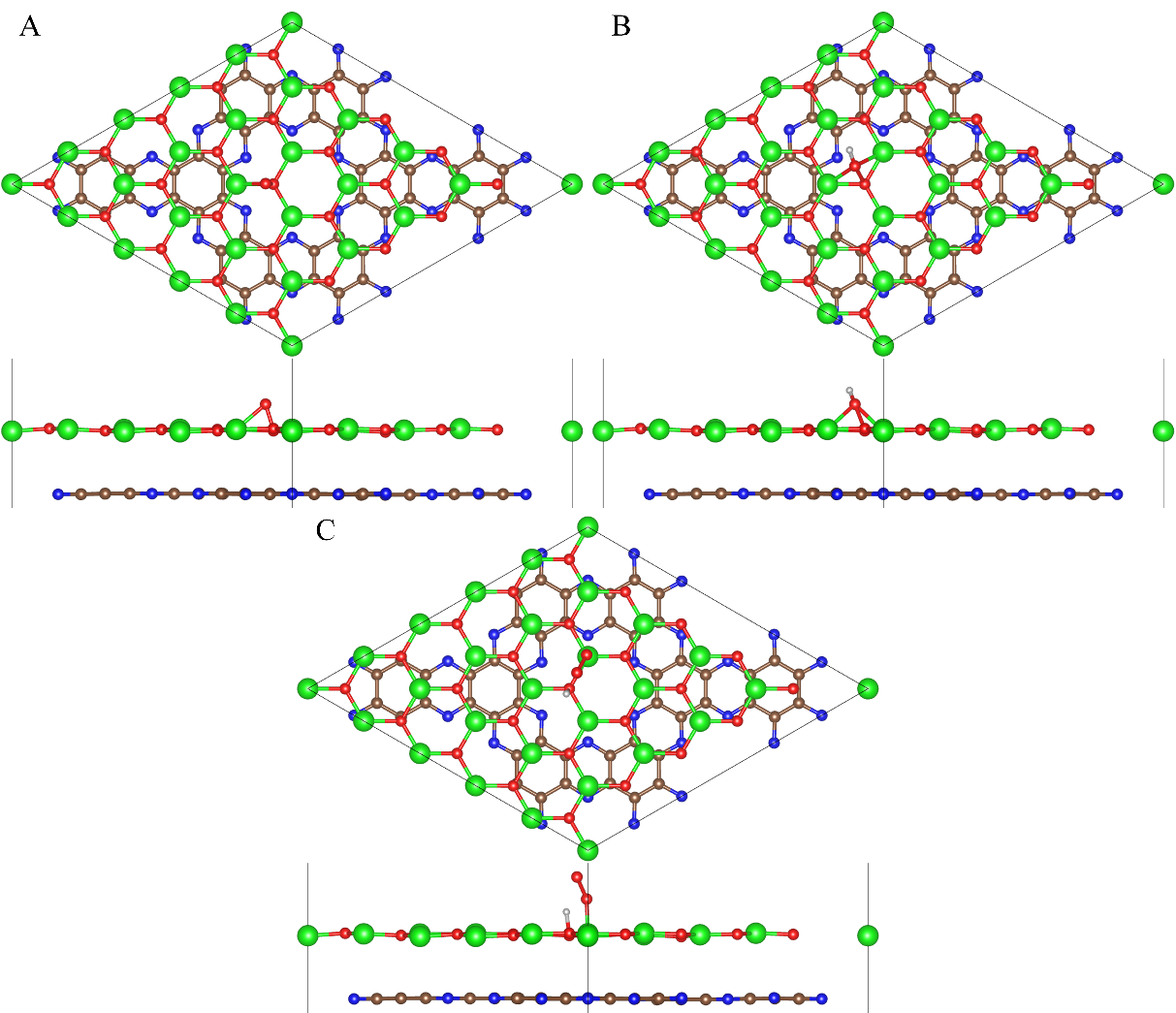


**Supplementary Figure S12**. The optimized geometries of (A) *O, (B) *OH, (C) *OOH intermediates.


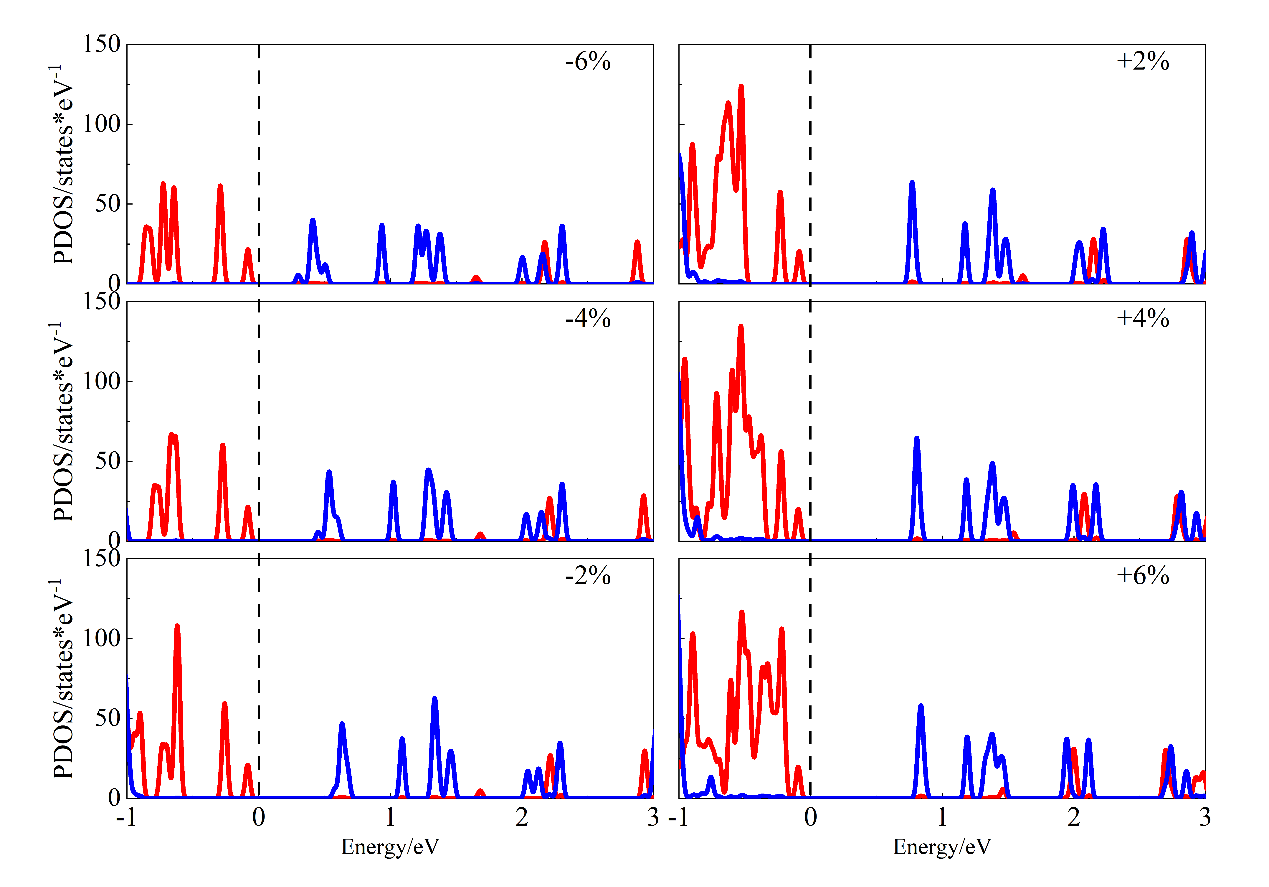


**Supplementary Figure S13**. The PDOS of ZnO/C_2_N heterostructure, based on PBE functional, with biaxial lateral strains. The red line line and blue line represent the contribution of ZnO and C_2_N, respectively.


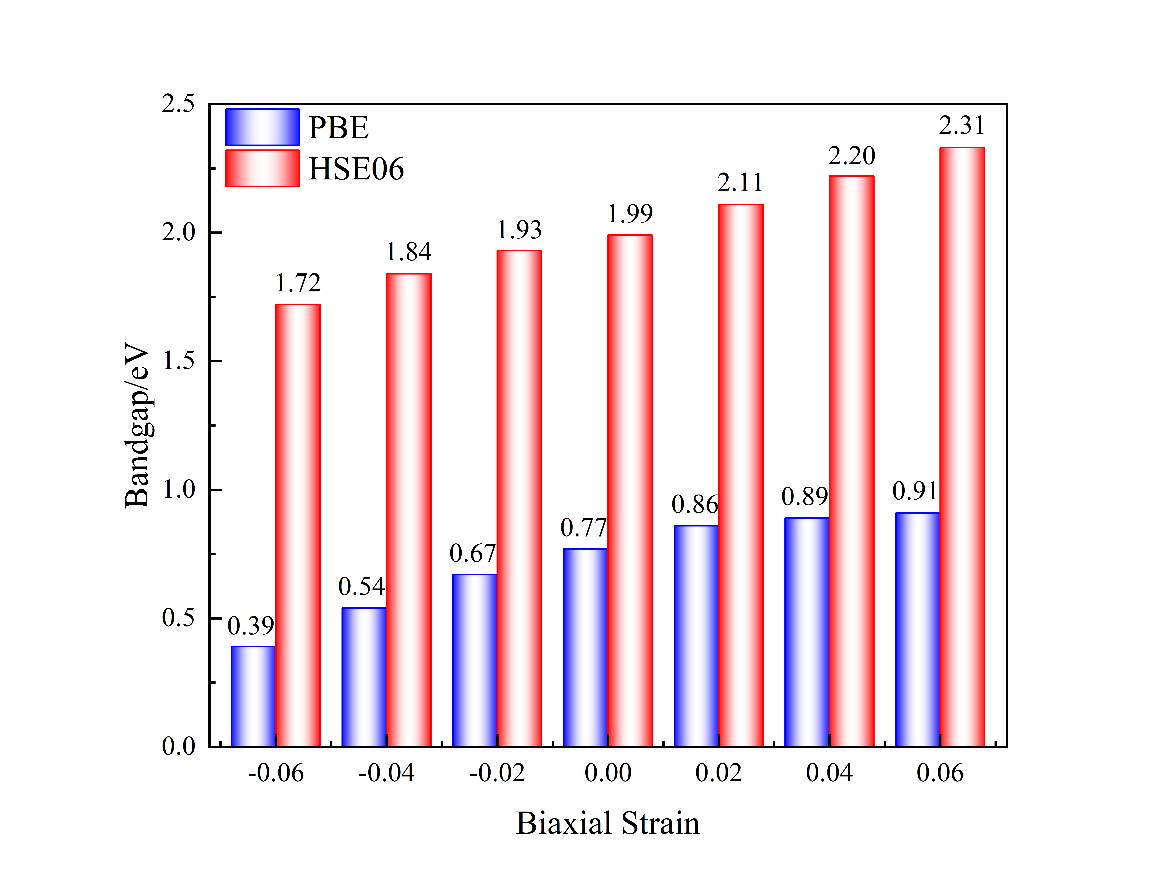


**Supplementary Figure S14**. Calculated band structures based on the HSE06 and PBE functional for strained ZnO/C_2_N heterostructure.


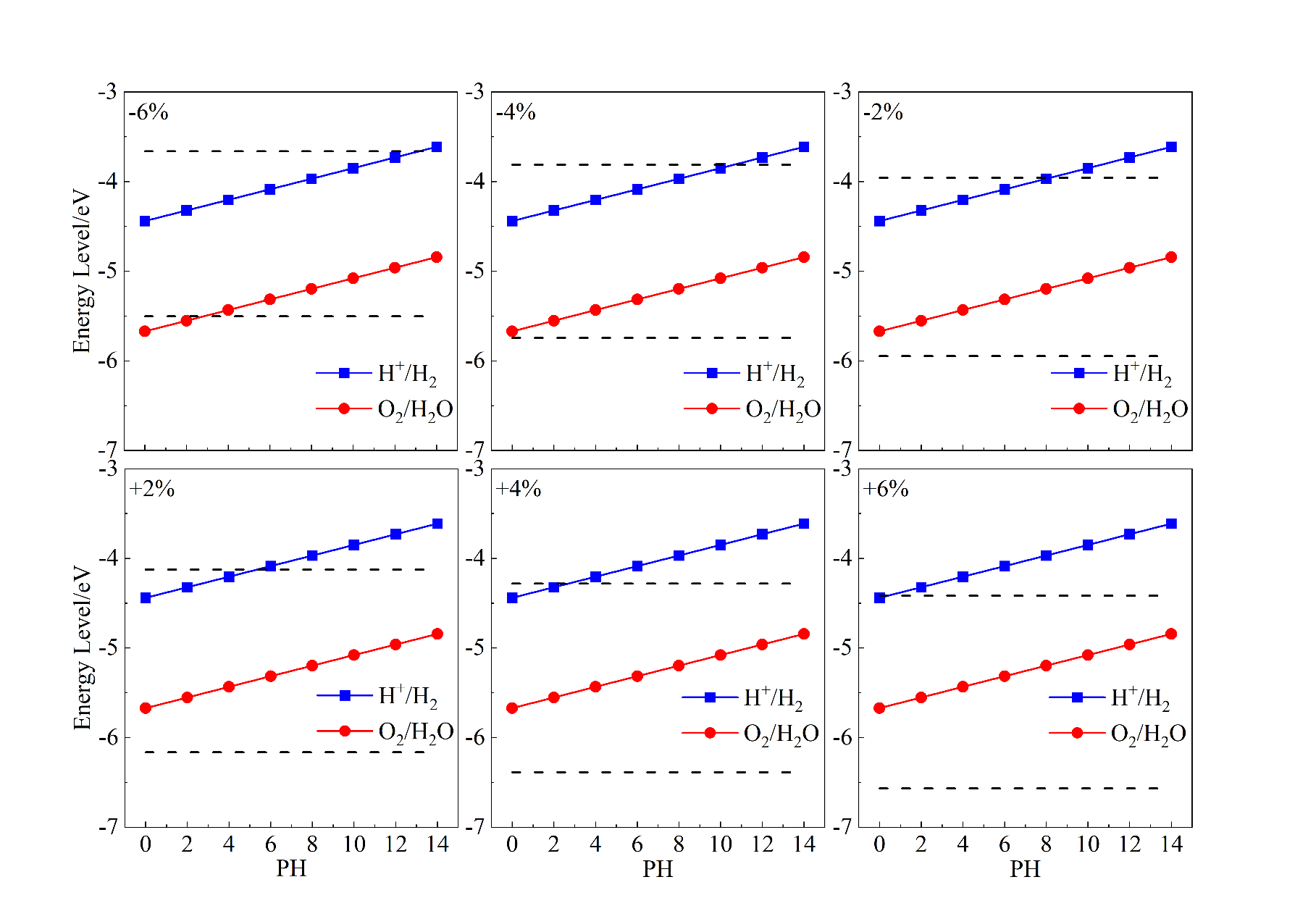


**Supplementary Figure S15**. Effect of strain on energy levels of VBM and CBM for ZnO/C_2_N heterostructures, compared with the redox potentials for water splitting in the absolute vacuum scale in different pH. The black dashed lines indicate the band edges of ZnO/C_2_N heterostructures.

Reference:

Bardeen, J., and Shockley, W. (1950). Deformation potentials and mobilities in non-polar crystals. *Phys. Rev.* 80(1)**,** 72. doi: 10.1103/PhysRev.80.72

Nørskov, J.K., Bligaard, T., Logadottir, A., Kitchin, J., Chen, J.G., Pandelov, S., et al. (2005). Trends in the exchange current for hydrogen evolution. *Journal of The Electrochem. Soc.* 152(3)**,** J23. doi: 10.1149/1.1856988

Skúlason, E., Tripkovic, V., Björketun, M.E., Gudmundsdóttir, S., Karlberg, G., Rossmeisl, J., et al. (2010). Modeling the electrochemical hydrogen oxidation and evolution reactions on the basis of density functional theory calculations. *J. Phys. Chem. C* 114(42)**,** 18182-18197. doi: 10.1021/jp1048887
